# An Abiotic Stress Responsive U-Box E3 Ubiquitin Ligase Is Involved in OsGI-Mediating Diurnal Rhythm Regulating Mechanism

**DOI:** 10.3390/plants9091071

**Published:** 2020-08-20

**Authors:** Yo-Han Yoo, Xu Jiang, Ki-Hong Jung

**Affiliations:** Graduate School of Biotechnology & Crop Biotech Institute, Kyung Hee University, Yongin 17104, Korea; directorhan@khu.ac.kr (Y.-H.Y.); kangwuk97@khu.ac.kr (X.J.)

**Keywords:** abiotic stress, diurnal regulation, OsGI, rice, U-box E3 ligase

## Abstract

The plant U-box (PUB) protein is the E3 ligase that plays roles in the degradation or post-translational modification of target proteins. In rice, 77 U-box proteins were identified and divided into eight classes according to the domain configuration. We performed a phylogenomic analysis by integrating microarray expression data under abiotic stress to the phylogenetic tree context. Real-time quantitative reverse transcription polymerase chain reaction (qRT-PCR) expression analyses identified that eight, twelve, and eight *PUB* family genes are associated with responses to drought, salinity, and cold stress, respectively. In total, 16 genes showed increased expression in response to three abiotic stresses. Among them, the expression of *OsPUB2* in class II and *OsPUB33*, *OsPUB39*, and *OsPUB41* in class III increased in all three abiotic stresses, indicating their involvement in multiple abiotic stress regulation. In addition, we identified the circadian rhythmic expression for three out of 16 genes responding to abiotic stress through meta-microarray expression data analysis. Among them, OsPUB4 is predicted to be involved in the rice GIGANTEA (OsGI)-mediating diurnal rhythm regulating mechanism. In the last, we constructed predicted protein-protein interaction networks associated with OsPUB4 and OsGI. Our analysis provides essential information to improve environmental stress tolerance mediated by the PUB family members in rice.

## 1. Introduction

Ubiquitination is a protein degradation system that regulates the amount of intracellular accumulation of signaling substances by selectively degrading certain proteins [1]. Especially in plants, various hormone signaling mechanisms, development, biotic, and abiotic stress signaling mechanisms have been reported to be closely related to ubiquitination [2,3]. Ubiquitin is a small 8-kDa protein in all eukaryotes. It is attached to specific proteins by the three enzymes Ub-activating enzyme (E1), Ub-conjugating enzyme (E2) and Ub-ligase (E3). Specific proteins that are subsequently polyubiquitinated are degraded by the 26S proteasome [4,5]. Among these, E3 ligase plays a major role in determining the specificity of the substrate and is largely divided into single-subunit E3 ligase and multi-subunit E3 ligase depending on the structure.

The single-subunit E3 ligase acts as an E3 ligase by itself, without any additional protein [6]. Single-subunit E3 ligase is subdivided into three proteins according to the domain: RING (for Really Interesting New Gene), U-box, and HECT (for Homology to E6-AP carboxyl terminus). Interestingly, other single-subunit E3 ligases exist in similar numbers among eukaryotes, but the number of U-box E3 ligases in plants is higher than in other eukaryotes. For example, 21 and 2 U-box E3 ligases were found in human and yeast [7,8], while Arabidopsis and rice were known to be 64 and 77, respectively [9,10]. Thus, the diversity of plant U-box genes suggests that the U-box domain may play an important role in performing plant-specific intracellular processes.

The circadian clock is an evolutionary system adapted to fluctuating environmental changes in the earth [11]. In particular, circadian clocks of immovable plants have more authority than animals and participate in various developmental processes [12]. The circadian clock is divided into an input that accepts the external environment signal, an oscillator that generates the rhythm according to the change cycle of the external environment, and an output that is controlled by this oscillator [13]. The most representative function of the output in the plant circadian clock is flowering. In other words, the circadian clock of the plant recognizes the photoperiod and determines the timing of the flowering [14]. For example, *Arabidopsis* induces the degradation of CYCLING DOF FACTOR 1 (CDF1), which prevents the expression of *CONSTANS* (*CO*), an important gene for flowering, by inducing the binding between two proteins of GIGANTEA (GI) and FLAVIN-BINDING, KELCH REPEAT, F-BOX 1 (FKF1) in the long-day condition. However, in a short-day condition, the binding between the two proteins GI and FKF1 is decreased, so that the expression of *CO* is kept low and the flowering time is delayed [15].

In rice, 77 U-box E3 ligase genes are known, of which only six genes have been reported [16,17,18,19,20]. In other words, many rice U-box E3 ligase genes have not yet been studied, and phenotypic interference due to functional redundancy may be one of the reasons [21]. Therefore, we analyzed transcriptome data using the phylogenomics tool and tried to obtain information on the environmental response characteristics of individual genes. This study comprehensively analyzed the expression characteristics in response to abiotic stress (drought, salinity, and cold) and the circadian clock which have not been studied in the previous genome-wide PUB family. Based on this, we will provide important fundamental data for studying the functions of individual *PUB* gene family.

## 2. Materials and Methods

### 2.1. Multiple Alignment and Phylogenetic Analysis

To perform our phylogenetic analysis of the PUB family, we used the protein sequences of 77 *PUB* genes identified in a previous global analysis of the rice PUB family [10]. The protein sequences for our phylogenomic analysis were downloaded from the Rice Genome Annotation Project Website [22]. After multiple-alignment of those sequences with ClustalX [23], we generated a phylogenetic tree using the Neighbor-Joining method, as incorporated in the MEGA5 tool kit for phylogenetic analysis [24].

### 2.2. Meta-Analysis of Gene Expression Data and Heatmap Development

Affymetric- and agilent-microarray data (GSE6901, GSE36040 and GSE38023), and RNA-seq data (GSE92989) were downloaded from the NCBI Gene Expression Omnibus (GEO, http://www.ncbi.nlm.nih.gov/geo/). We then uploaded the normalized expression data to the Multi Experiment Viewer and visualized the data via heatmaps (http://www.tm4.org/mev.html).

### 2.3. Plant Materials and Abiotic Stress Treatment

Rice (*O. sativa* L.cv. Dongjin) seeds were germinated on the Murashige Skoog medium for 14 days at 28 °C. Subsequently, the seedlings were washed with sterilized water to completely remove the agar and were air-dried for 0, 2, 6, and 12 h at 28 °C for drought stress treatment [25]. To simulate salinity stress, we exposed 14-day-old plants to 200 mM NaCl for 0, 2, 6, and 12 h at 28 °C [26]. In the last, we exposed 14-day-old plants to 4 °C ± 1 °C for 0, 2, 6, and 12 h for cold stress treatment. The control plants remained at 28 °C. Leaves and roots of three plants were pooled for one biological replicate and each treatment had three biological repeats.

### 2.4. Plant Materials for Diurnal Rhythm

To investigate the functional associations of PUB family members with the diurnal rhythm and *OsGI (GIGANTEA)*-mediating regulatory pathway, Wild-type (WT) plant and *osgi* mutant seeds (*LOC_Os01g08700*) were germinated on the Murashige Skoog medium for 7 days at 28 °C [27]. They were then transferred to individual pots and grown in an incubator (12-h light/12-h dark, 28 °C/22 °C) for 30 days. After that, their leaves were sampled at 2-h intervals for 24 h.

### 2.5. RNA Extraction and Real-Time Quantitative PCR

Samples were frozen in liquid nitrogen and total RNAs were extracted using RNAiso Plus (Takara Bio, Kyoto, Japan). Using MMLV Reverse Transcriptase (Promega, Madison, WI, USA) and the oligo(dT) 15 primer, first-strand cDNA was synthesized as we recently reported [28,29]. For normalizing the amplified transcripts, we used a primer pair for rice *ubiquitin 5* (*OsUbi5/Os01g22490*) [30]. All primers for these analyses are summarized in Table S1.

### 2.6. Analysis of a Predicted Protein–Protein Interaction Network

Using the STRING tool (https://version-10-5.string-db.org/) [31], we generated a hypothetical protein–protein interaction network involving E3 ubiquitin ligase, transcription factors (TFs), and flowering regulatory genes. The network was edited with the Cytoscape tool (https://cytoscape.org/; version 3.6.0) (The Cytoscape Consortium, New York, NY, USA) [32,33].

## 3. Results and Discussion

### 3.1. Integration of Abiotic Stress Expression Patterns with a Phylogenetic Tree Context of the Rice PUB Family Reveals the Key PUB Family Members for the Stress Responses

**Figure 1:**
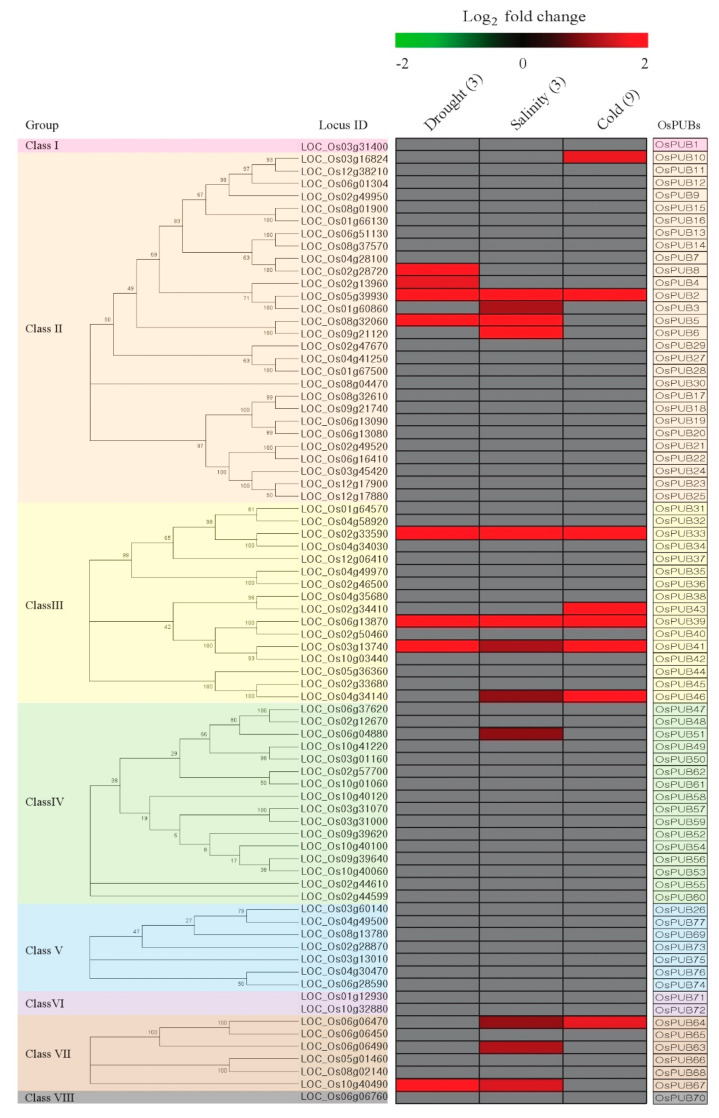
Meta-analysis of *OsPUB* genes expression patterns using drought- salinity- and cold-treated microarray data. On the left side of the heat-map is a phylogenetic tree for each class. Green, low level of log_2_ intensity; red, high level; gray, *t*-test *p*-value > 0.01 or enrichment values of <1 (log_2_)-fold.

According to the recent report on the PUB family in rice, 77 estimated U-box proteins were identified through a whole-genome analysis algorithm and divided into eight classes according to the domain configuration (Figure 1 and Figure S1) [10]. We have constructed a phylogenetic tree using protein sequences for each of the five classes except I, VI, and VIII, which have only one or two genes in the eight classes. The expression of 77 genes was visualized using drought- and salinity-treated RNA-seq data (GSE92989) using seedling roots [26], and cold-treated microarray data (GSE6901 and GSE38023) from using seedling leaves. When the resultant microarray data were examined according to criteria where *t*-test *p*-values were <0.01 and upregulation showed a greater than 1 (log_2_ scale)-fold change for control versus abiotic stress, we were able to identify 16 genes (Figure 1). The remaining genes in the heat-map were visualized in gray color. As a result, we identified eight (*OsPUB2*, *OsPUB4*, *OsPUB5*, *OsPUB8*, *OsPUB33*, *OsPUB39*, *OsPUB41* and *OsPUB67*), twelve (*OsPUB2*, *OsPUB3*, *OsPUB5*, *OsPUB6*, *OsPUB33*, *OsPUB39*, *OsPUB41*, *OsPUB46*, *OsPUB51*, *OsPUB63*, *OsPUB64* and *OsPUB67*), and eight (*OsPUB2*, *OsPUB10*, *OsPUB33*, *OsPUB39*, *OsPUB41*, *OsPUB43*, *OsPUB46* and *OsPUB64*) upregulated genes under drought, salinity, and cold stress conditions [25,26]. Interestingly, expression of *OsPUB2* in class II and *OsPUB33*, *OsPUB39*, and *OsPUB41* in class III increased in all three abiotic stresses. In addition, most genes with increased expression in drought, salinity, and cold stress were included in classes II, III, and VII. These results indicate that among the 77 PUB families, there are pivotal classes and genes that respond to environmental stress.

### 3.2. Real-Time Quantitative PCR Analysis Confirmed Expression Patterns in Response to Drought, Salt and Cold Stresses of 16 PUB Family Genes in Rice

To confirm the global transcriptome data, we carried out quantitative reverse transcription polymerase chain reaction (qRT-PCR) analysis of PUB family genes in the drought, salinity, and cold stress conditions. Fourteen-day-old seedlings were treated with drought, salinity, and cold stress for 0, 2, 6, and 12 h, respectively. Root samples under drought and salinity stress and leaf samples under cold stress are collected for cDNA synthesis.

**Figure 2:**
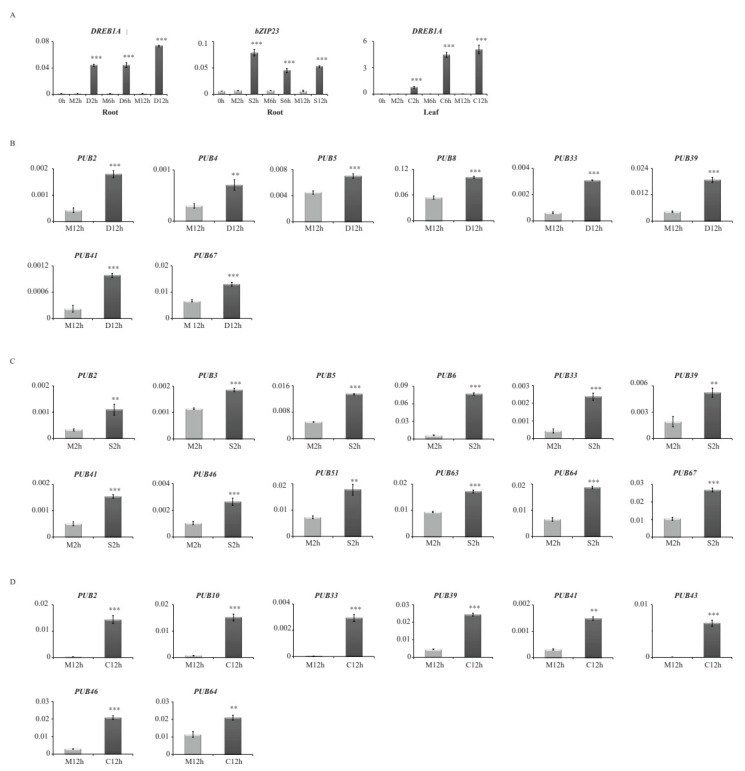
qRT expression profiles for 16 *OsPUB* genes selected from global transcriptome data analysis. OsDREB1A and OsbZIP23 were used as marker genes for abiotic stress (**A**). Abiotic stress samples were prepared from drought (**B**), salinity (**C**), and cold (**D**) (0, 2, 6, and 12 h) in root or leaf. Rice ubiquitin (*OsUbi5*) was served as internal control. ** *p* value < 0.01; *** *p* value < 0.001. N = 3.

As the first step, we tested the expression of *OsDREB1A*, a drought and cold stress marker gene, for drought (0, 2, 6, and 12 h) and cold (0, 2, 6, and 12 h) stress samples, and of expression of *OsbZIP23*, a salt stress marker gene, for salt (0, 2, 6, and 12 h) stress samples [34,35]. As expected, expressions of *OsDREB1A* and *OsbZIP23* were significantly stimulated compared to the control at all tested time points of stress treatment (Figure 2), indicating that samples under drought, salt, and cold stress treatments are well qualified for the further differential expression analyses. To validate expression patterns of selected PUB genes, we chose a 2 h sample (salinity) and 12 h samples (drought and cold) out of the time series stress treatments showing stronger and more stable upregulation of marker genes. Subsequently, we confirmed that expressions of 16 PUB genes were significantly upregulated under drought, salinity and cold stresses (Figure 2). These results are in agreement with expression patterns analyzed using transcriptome data associated with abiotic stress.

### 3.3. PUB Family Genes Are Involved in OsGI Mediating Diurnal Regulation Pathway

**Figure 3:**
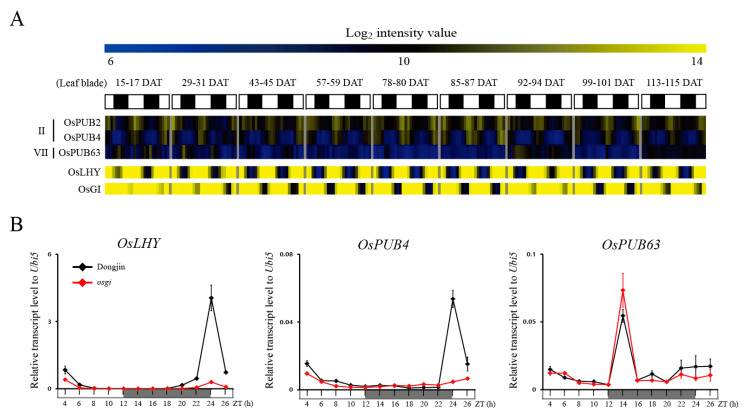
Diurnal expression patterns of two *OsPUB* genes in mature leaves, using available Agilent 44k array data over the entire plant life span (**A**), or evaluated at 12 time points over 24-h period in “Dongjin” rice and *osgi* mutant (**B**). *OsLHY* was standard marker gene for diurnal rhythm. *OsUbi5* was served as the internal control. The continuous white and black bars indicate day and night time, respectively. ZT, zeitgeber time (ZT = 0 at lights-on).

Diurnal rhythm in plants is regulated by light and the circadian clock, and metabolism, physiology, and behavior change between day and night [36]. In addition, recent studies have shown that circadian rhythm correlates with abiotic stress [37,38,39]. To identify the *PUB* genes in rice associated with diurnal rhythm, we analyzed expression patterns using publicly available Agilent 44k array data (GSE36040) obtained in rice leaves harvested under diurnal rhythm in nine different developmental stages [40]. Of the 77 *PUB* genes, nine (*OsPUB2*, *OsPUB4*, *OsPUB16*, *OsPUB20*, *OsPUB34*, *OsPUB47*, *OsPUB52*, *OsPUB63*, and *OsPUB77*) genes were observed to show diurnal rhythm in the leaves (Figure S1). Among the 16 genes with increased expression in Abiotic stress, three (*OsPUB2*, *OsPUB4*, and *OsPUB63*) genes were associated with diurnal rhythm (Figure 1). To confirm expression associated with diurnal rhythm, 37-day-old leaves were sampled at 2 h intervals for 24 h, and we confirm that diurnal rhythms of *OsPUB4* and *OsPUB63* were observed through Real-Time qPCR analyses. There was no difference in expression in the dark state of *OsPUB4*, but expression increased when the plant first recognized the light (Figure 3). In contrast, *OsPUB63* showed no expression difference in the light state but increased expression in the dark state (Figure 3). Unfortunately, *OsPUB2* was associated with diurnal rhythms in the Agilent 44k array data, but no significant change in expression was observed in Real-Time qPCR analysis (Figure S2).

To obtain the insight into the mechanism on the regulation of the diurnal rhythm of these PUB genes, we used rice *gi* mutants with defects in the diurnal rhythm [27]. In *osgi*, diurnal expression of a well-known marker gene for diurnal rhythm, *LATE ELONGATED HYPOCOTYL (LHY)* [41], was dramatically down-regulated across all time points (Figure 3B). Interestingly, *OsPUB4*, like *LHY*, disappeared from diurnal rhythm expression patterns in *osgi* mutants (Figure 3B). In contrast, *OsPUB63* was able to observe the same diurnal rhythm expression patterns in both the control (dongjin) and *osgi* mutants. These results indicate that the *OsPUB4* gene is involved to the *OsGI*-mediating diurnal rhythm regulating mechanism.

### 3.4. OsPUB4 Is Under the Control of OsGI, One of Main Regulators of the Circadian Clock

**Figure 4:**
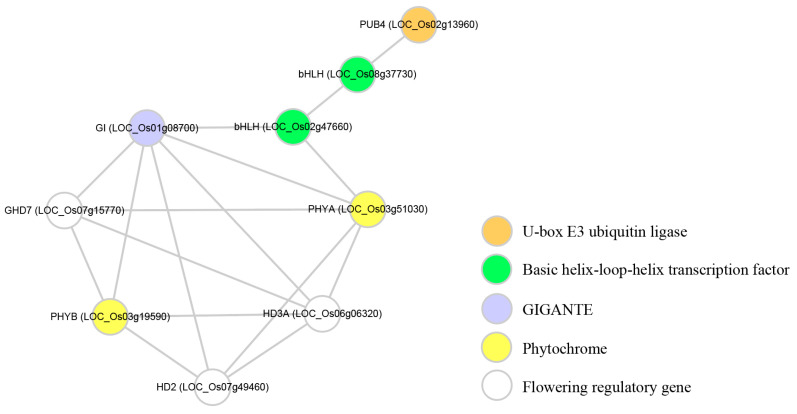
Construction of predicted protein-protein interaction networks associated with OsPUB4 and OsGI. Using the STRING tool, we found seven proteins that are expected to interact with OsPUB4 (orange circle) and OsGI (blue circle). Three of the seven proteins were identified as flowering regulatory genes (White circles), two as phytochrome (yellow circles), and two as bHLH TF (green circles).

GI is involved in maintaining the circadian clock of downstream genes. It has been reported that the circadian rhythms of flowering regulatory genes such as *Ehd1* (*Early heading date 1*), *Hd3a* (*Heading date 3a*), *RFT1* (*RICE FLOWERING LOCUS T 1*), *Hd1* (*Heading date 1*) and *OsMADS51* are significantly reduced in *osgi* mutants [27,42,43]. Interestingly, like the flowering regulation genes mentioned earlier, the circadian rhythm of *OsPUB4* also decreases in the *osgi* mutant (Figure 3). This result indicates that OsPUB4 might be under the control of OsGI, one of the main regulators of the circadian clock. We created a putative network on the STRING website (https://string-db.org/cgi/input.pl?sessionId=dsUIDFue7qrX&input_page_show_search=on) to check the correlation between OsPUB4 and OsGI. As expected, the network includes photoreceptors such as PHYA (Phytochrome A), PHYB (Phytochrome B), and transcription factors related to flowering time such as Ghd7 (Grain number, plant height, and heading date7), HD3A and HD2 (Figure 4).

## 4. Conclusions

In this study, we selected genes that respond to abiotic stress in the *OsPUB* family, and further confirmed their circadian clock. Recent studies have shown that genes associated with the circadian clock and flowering time are associated with abiotic stress [38,39]. For example, in Arabidopsis, the *GI*-overexpressed transgenic plants show increased salt sensitivity, while the *osgi* mutants show a salt tolerance phenotype [44]. In addition, *LOV KELCH protein 2* (*LKP2*), which regulates circadian rhythm and flowering time in plants, increases dehydration tolerance when overexpressed [45]. The key clock component (TOC1, the timing of CAB expression 1) that binds to the promoter of the ABA-related gene increases drought tolerance in *toc1-RNAi* plants. Conversely, overexpression of *TOC1* increases water loss in drought conditions, leading to a decrease in survival rate [46]. Interestingly, there is no correlation between the effects of mutations on clock function and abiotic stress resistance. Instead, changes in the expression level of the clock gene in the mutants are presumed to have a direct effect on the regulation of the abiotic stress response [38]. For example, half of the genes responsive to drought, salinity, heat, and osmoticum were found to have diurnal rhythm [47]. This transcriptomic analysis suggests that many genes that respond to abiotic stress are under the control of the circadian clock.

Therefore, we speculate that OsPUB4 will play a role similar to COP1 (Constitutive photomorphogenic 1). COP1 is an E3 ubiquitin ligase containing RING-finger and WD40 domains, and is known to be involved in the control of seedling development, flowering time, and circadian rhythm [48]. In particular, HY5 (Long hypocotyl 5), PHYA, PHYB, PIL1 (Phytochrome interacting factor 3-like 1), CO (CONSTANS), GI (GIGANTEA), etc. were identified as substrates of COP1 [49,50,51,52,53,54]. Accordingly, we expect OsPUB4 to participate in circadian rhythms and abiotic stress responses by controlling the stability of various proteins.

## Supplementary Materials

The following are available online at https://www.mdpi.com/2223-7747/9/9/1071/s1, Figure S1: Heat-map expression data associated with the diurnal rhythm of the *OsPUB* family genes. Figure S2: Diurnal expression patterns of *OsPUB2* gene evaluated at 12 time points over 24-h period in “Dongjin” rice and *osgi* mutant. Table S1: Summary of primer sequences used for qRT-PCR analyses of this study.
